# Motor expertise modulates unconscious rather than conscious executive control

**DOI:** 10.7717/peerj.6387

**Published:** 2019-02-05

**Authors:** Fanying Meng, Anmin Li, Yihong You, Chun Xie

**Affiliations:** Department of Sport Psychology, School of Kinesiology, Shanghai University of Sport, Shanghai, China

**Keywords:** Motor expertise, Unconscious executive control, Conscious executive control, Table tennis athletes

## Abstract

**Background:**

Executive control, the ability to regulate the execution of a goal-directed task, is an important element in an athlete’s skill set. Although previous studies have shown that executive control in athletes is better than that in non-athletes, those studies were mainly confined to conscious executive control. Many recent studies have suggested that executive control can be triggered by the presentation of visual stimuli without participant’s conscious awareness. However, few studies have examined unconscious executive control in sports. Thus, the present study investigated whether, similar to conscious executive control, unconscious executive control in table tennis athletes is superior to that in non-athletes.

**Methods:**

In total, 42 age-matched undergraduate students were recruited for this study; 22 nonathletic students lacking practical athletic experience comprised one group, and 20 table tennis athletes with many years of training in this sport comprised a second group. Each participant first completed an unconscious response priming task, the unconscious processing of visual-spatial information, and then completed a conscious version of this same response priming task.

**Results:**

Table tennis athletes showed a significant response priming effect, whereas non-athletes did not, when participants were unable to consciously perceive the visual-spatial priming stimuli. In addition, the number of years the table tennis athletes had trained in this sport (a measure of their motor expertise) was positively correlated with the strength of the unconscious response priming effect. However, both table tennis athletes and non-athletes showed a response priming effect when the primes were unmasked and the participants were able to consciously perceive the visual-spatial priming stimuli.

**Conclusion:**

Our results suggest that motor expertise modulates unconscious, rather than conscious, executive control and that motor expertise is positively correlated with unconscious executive control in table tennis athletes.

## Introduction

Executive control refers to the ability to regulate the execution of a goal-directed task (De Pisapia, 2013; McBride et al., 2012). It can be decomposed into three subprocesses: updating representations in working memory, inhibiting prepotent responses and switching between tasks or mental sets (Miyake et al., 2000). Executive control helps athletes to make decisions in a short time under mental pressure, thus it is regarded as an important element in an athlete’s skill set that predicts their success (Vestberg et al., 2012; Vestberg et al., 2017). Previous research has extensively studied the relationship between motor expertise and executive control, and indicate that the executive control can benefit from motor expertise in athletes, in sports such as soccer (Verburgh et al., 2014), badminton (Yu et al., 2017), basketball (Alarcon et al., 2017) and fencing (Chan et al., 2011). However, these views are mainly based on studies that examined executive control elicited by stimuli that are consciously perceived, which we define as conscious executive control. In the studies mentioned above, the amount of time allotted is not entirely consistent with performance in a real sport. Given the time pressure in real sport scenarios, athletes, especially those with expertise in open skill sports (e.g., table tennis, tennis, badminton), tend to make motor responses to a given situation without consciously processing due to the rapid changes of the external environment (Kibele, 2006). Recent studies have shown that executive control can be triggered by visual stimuli without a participant’s awareness (Boy, Husain & Sumner, 2010; Ulrich & Kiefer, 2016; Van Gaal et al., 2011), which we here define as unconscious executive control. However, the unconscious executive control in sports has largely been ignored. Hence, the focus of the present study was to investigate whether athletes have better performance than non-athletes in unconscious executive control.

Many researchers suggest that unconscious executive control is a common phenomenon (Hommel, 2008; Prabhakaran & Gray, 2012; Suhler & Churchland, 2009). The masked priming task is one of the most widely used paradigms to study unconscious processing in executive control (Dehaene et al., 1998; Kiefer, 2012; Kiefer & Martens, 2010). In this task, prior to the target, a stimulus known as prime that required the same or different response with target was presented. The prime is briefly presented and mask is used to prevent from the prime to be perceived consciously. Participants are instructed to make a response to target with a left or right key press. Under the congruent condition, both prime and target activated the same response, which leads to faster responses. By contrast, the prime and target activated the different response under the incongruent condition, which leads to response conflict. Typically, participants respond faster and commit fewer errors in the congruent condition than in the incongruent condition. This is also known as unconscious response priming effect (Avneon & Lamy, 2018; Kunde, 2004), which is regarded as an indirect measure to assess the particular process that involved in unconscious executive control (Ansorge, Kunde & Kiefer, 2014).

Although results from studies that have investigated unconscious response priming with a masked priming paradigm in typists (Heinemann et al., 2010), chess players (Kiesel et al., 2009), and college students engaged in familiar tasks (Reuss et al., 2015) suggest that practice or expertise may be the important prerequisite for unconscious executive control. There were only two studies that investigate the relationship between motor expertise and unconscious processing in sport. Gueldenpenning et al. (2011) used a masked priming task to investigate whether motor expertise modulate the unconscious processing of human body postures, the results indicated that both high jump athletes and non-athletes did not exhibit unconscious response priming effect. However, another study conducted Gueldenpenning et al. (2015) with similar task reported that martial arts athletes outperform novices and show the unconscious response priming effect when their targets change from a front to a back perspective. In view of the inconsistent results, the modulatory effect of specific motor experience on unconscious processing in executive control need to be further defined.

According to the theory of event coding put forward by Hommel et al. (2001), athletes’ superior in executive control is because perceived events (perceptions) and to-be-generated events (actions) are commonly coded, specific motor experience is necessary during this processing. Compared to non-athletes, athletes with specific motor experience show higher perceptual sensitivity (Schütz-Bosbach & Prinz, 2007). Gueldenpenning et al. (2011) suggested that high perceptual sensitivity in the domain of expertise might also be the important prerequisite for unconscious executive control. Therefore, athletes whom show superior conscious executive control may also show superior unconscious executive control.

To address these questions, the present study investigated whether motor expertise modulates unconscious (experiment 1) and conscious (experiment 2) executive control. Thus, table tennis athletes and aged-matched college students without athletic expertise were recruited to complete an unconscious and conscious version of the same priming task, in which the masked stimuli can be manipulated so that the primes are masked (unconscious) or unmasked (conscious) (Jiang et al., 2016). The reasons for choosing table tennis athletes as the participants are as follows: Firstly, table tennis is considered an open skill sport that is associated with stronger executive control (Wang et al., 2013; Yu et al., 2017) and more unconscious behaviors (Kibele, 2006; Raab & Johnson, 2008) than are closed skill sports. In addition, table tennis athletes usually execute shots unconsciously in training or competitions because of the high speed of the ball and the close distance between opponents, which may lead to better unconscious executive control. Lastly, table tennis is one of the most popular sports in China, table tennis athletes have a high competition level because of the systematical training (Wang, Guo & Zhou, 2016), which are the perfect samples to investigate the relationship between motor expertise and unconscious executive control. We hypothesized that athletes have better performance than non-athletes in executive control both on the conscious and unconscious level.

## Experiment 1

Experiment 1 investigated whether motor expertise modulates unconscious processing of visual-spatial information in a group of table tennis athletes and a group of non-athletes. On the basis of previous findings that expertise may be an important prerequisite for unconscious information processing (Heinemann et al., 2010; Kiesel et al., 2009), we hypothesized that motor expertise would modulate the unconscious processing of visual-spatial information; thus, we posited that table tennis athletes would respond faster and commit fewer errors on congruent trials than incongruent trials, whereas non-athletes would show no significant difference in response times and errors between these two conditions.

### Methods and materials

#### Ethical approval

This study received approval from the Ethics Committee of Shanghai University of Sport (No. 2018025).

#### Participants

Forty-two participants participated voluntarily and received financial compensation after completing all the experiments. In order to find an interaction between expertise and response congruency in line with You et al. (2018) power analysis (G*Power 3.1, *α* = 0.05, power = 0.80, effect size = 0.24) showed that a minimum 16 volunteers needed to participate. In total, 42 age-matched volunteers were recruited for this study. We assigned 22 college students (seven women; mean age 20.27 years, ranging from 18 to 25 years) to the non-athlete group because they reported having had no practical experience with table tennis or other racket games. We assigned 20 age-matched table tennis athletes to the athlete group (six women; mean age 20.55 years, ranging from 18 to 23 years) because of their extensive practical experience in table tennis. All table tennis athletes had gained the first or second level of the national standard. The mean training experience for the athlete group was 6.8 years and their mean training frequency was 3.72 times per week. All participants were healthy, were right-handed, and had normal or corrected to normal vision. All participants provided written informed consent before being tested.

#### Stimuli

For table tennis players, rapidly judging where and when to hit the ball (i.e., the hitting point) and responding correctly are the key factors that determine the outcome of competitions. We designed the stimuli used in present study according to the item characteristics of table tennis based on the type–token model. The type–token model, proposed by Ecker, Groh-Bordin & Zimmer (2004), suggests that perceptual priming and episodic recognition are mainly based on three kinds of representations, namely, type traces, object tokens, and episodic tokens. Later, Zimmer & Ecker (2010) characterized these representations as types and tokens. *Types* refer to the prototypical representation, including the outline and three-dimensional information that are essential for object recognition. *Tokens* support episodic recognition, such as orientation and color information. Tokens can bind with types and be stored in memory in a simplified form when individuals are familiar with the presented information (Zimmer & Ecker, 2010). Thus, we designed circles with notches at angles of 45°, 135°, 225°, or 315° as primes and targets; the circle provided the outline information, and the notch appearing at different points on the circle provided information about the hitting point. The combination of outline and hitting point information can prime the cognitive processes associated with the motor expertise for table tennis athletes. In our previous study, these stimuli were selected as experience-related stimuli to investigate the mechanism of visual-spatial information processing in table tennis athletes, and that study showed that compared with non-athletes, table tennis athletes had better performance, exhibited higher functional coupling and neural efficiency when judging notches in circles (Guo, Li & Wang, 2015; Guo, Li & Yu, 2017).

#### Tasks

A Lenovo computer with a 17-inch VGA display (frequency 60 Hz, resolution 1366 × 768) was used for stimulus presentation, and the E-prime 2.0 software package (Psychology Software Tools, Pittsburgh, PA, USA) was used for response sampling. All stimuli were presented on a gray background with a size of 6 × 6 cm and subtended a visual angle of 5.73° horizontally and 5.73° vertically from a viewing distance of 60 cm.

##### Unconscious priming task.

Participants were instructed to decide the notch orientation (angle) on circles of the presented targets as quickly and accurately as possible by pressing the corresponding key on the computer keyboard. A circle with a notch at an angle of 225° or 315° was assigned to the “f” key, and a circle with a notch at an angle of 45° or 135° was assigned to the “j” key. To reduce the visibility of the prime in this unconscious priming response task, the prime was presented for 33 ms, and masking patterns were added before and after the prime presentation.

**Figure 1:**
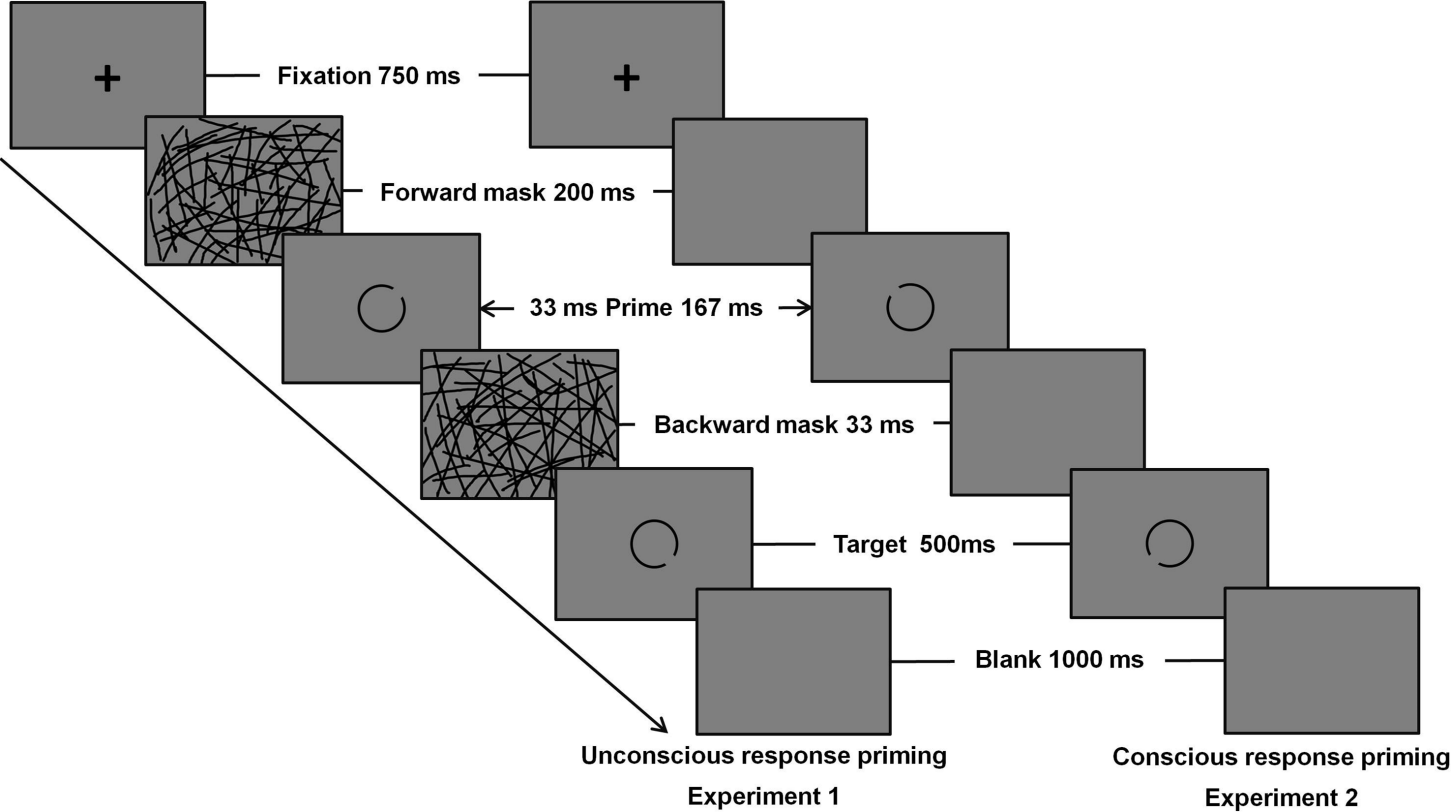
The unconscious and conscious priming task. Sequence within a single trial of the unconscious priming task in experiment 1 and conscious priming task in experiment 2.

Each trial started with the presentation of a fixation cross for 750 ms, followed by a forward mask (a picture containing many randomly oriented lines) that was presented for 200 ms. After the forward mask, the prime appeared and lasted for 33 ms. When the prime disappeared, a backward mask (another picture containing many randomly oriented lines) was shown for 33 ms. The backward mask was replaced by the target for 500 ms. Then, a blank screen was shown for 1,000 ms (Fig. 1). Participants had to report their decision within 1,500 ms, otherwise the next trial would be triggered. The interval before the next trial started lasted randomly from 1,000 to 1,500 ms.

Each participant performed a practice block of 24 trials, which included all eight possible prime-target combinations in a randomized order. The unconscious priming task followed and consisted of 160 experimental trials that were divided into four blocks. Within each block, half of the trials were congruent trials, that is, the prime and target had same response. The remaining were incongruent trials, in which the prime and target had different response. The four circles with notches at one of four different angles appeared equally often as primes and targets and were varied across trials to avoid repetition priming. Participants were not told of the existence of the prime before or during this experiment.

##### Prime identification task.

To assess prime visibility, an objective measure of prime identification was performed after completing the unconscious priming task. Participants were informed about the existence of the primes. This task included 64 trials, and the procedure was identical to the unconscious priming task. The masked prime had the same or a different response to the subsequently presented target. Participants had to discriminate the notch angle of the prime without any time pressure. No feedback was given during this task.

### Statistical analysis

#### Identification rates

The signal detection measure *d*′ was used to assess the visibility of the primes (Green & Swets, 1966). The hit rates (correct response to target on congruent trials) and false-alarm rates (erroneous response to target on incongruent trials) were calculated for each participant (Kiefer & Martens, 2010). One sample *t*-tests were performed to compare the difference between the identification rate *d*′ and the chance level of 50% for table tennis athletes and non-athletes respectively. Independent *t*-test was performed to compare the identification rate *d*′ between table tennis athletes and non-athletes.

In addition, the distribution of the strength of the unconscious response priming (Kolmogorov–Smirnov = 0.11, *p* = 0.20) was normal. However, distribution of the averaged *d*′ (Kolmogorov–Smirnov = 0.14, *p* = 0.045) was not normal. Hence, a logarithmic conversion is applied to the averaged *d*′ to meet the prerequisite of correlation analysis. The Pearson’s correlation was performed to explore the relationship between the strength of the unconscious response priming effect and the identification rate *d*′. The strength of unconscious response priming effect was expressed as the reaction time on incongruent trials minus the reaction time on congruent trials when the prime was masked (Kunde, 2004).

#### Reaction times and response errors

Reaction times (RTs) that deviated by more or less than two standard deviations (3.53%) and incorrect trials and missed trials (4.39%) were rejected from further analysis. The mean RT of the correct responses and the mean error rate (ER) were calculated for each participant and each experimental condition (congruent vs. incongruent trials). Mean RT and ER were examined separately in two-way repeated measures analysis of variance (ANOVA), with the within-subjects factor as response congruency (congruent vs. incongruent) and the between-subjects factor as expertise (athletes vs. non-athletes).

#### Correlation analysis

The distribution of the strength of the unconscious response priming effect (Kolmogorov–Smirnov = 0.17, *p* = 0.14) and the number of years of training (Kolmogorov–Smirnov = 0.18, *p* = 0.08) were normal. The correlation between the number of years of training and the strength of the unconscious response priming effect in table tennis athletes was assessed by Pearson’s correlation.

### Results

#### Prime visibility

The discrimination measure *d*′  was −0.04 for table tennis athletes, which did not deviate significantly from zero (*t*_(19)_ =−0.24, *p* = 0.81), and *d*′  was −0.15 for non-athletes, which also did not significantly deviate from zero, (*t*_(21)_ = −1.43, *p* = 0.17). The *d*′  values of table tennis athletes and non-athletes did not differ significantly, (*t*_(40)_ = 0.57, *p* = 0.58).

In addition, the strength of the unconscious response priming effect and individual averaged *d*′  did not correlate with each other (*r*_(42)_ = 0.04, *p* = 0.83), which suggested that the unconscious response priming effect was not the result of participants’ awareness of masked primes. These results indicate that the participants could not consciously perceive the prime.

#### Reaction times and response errors

**Figure 2:**
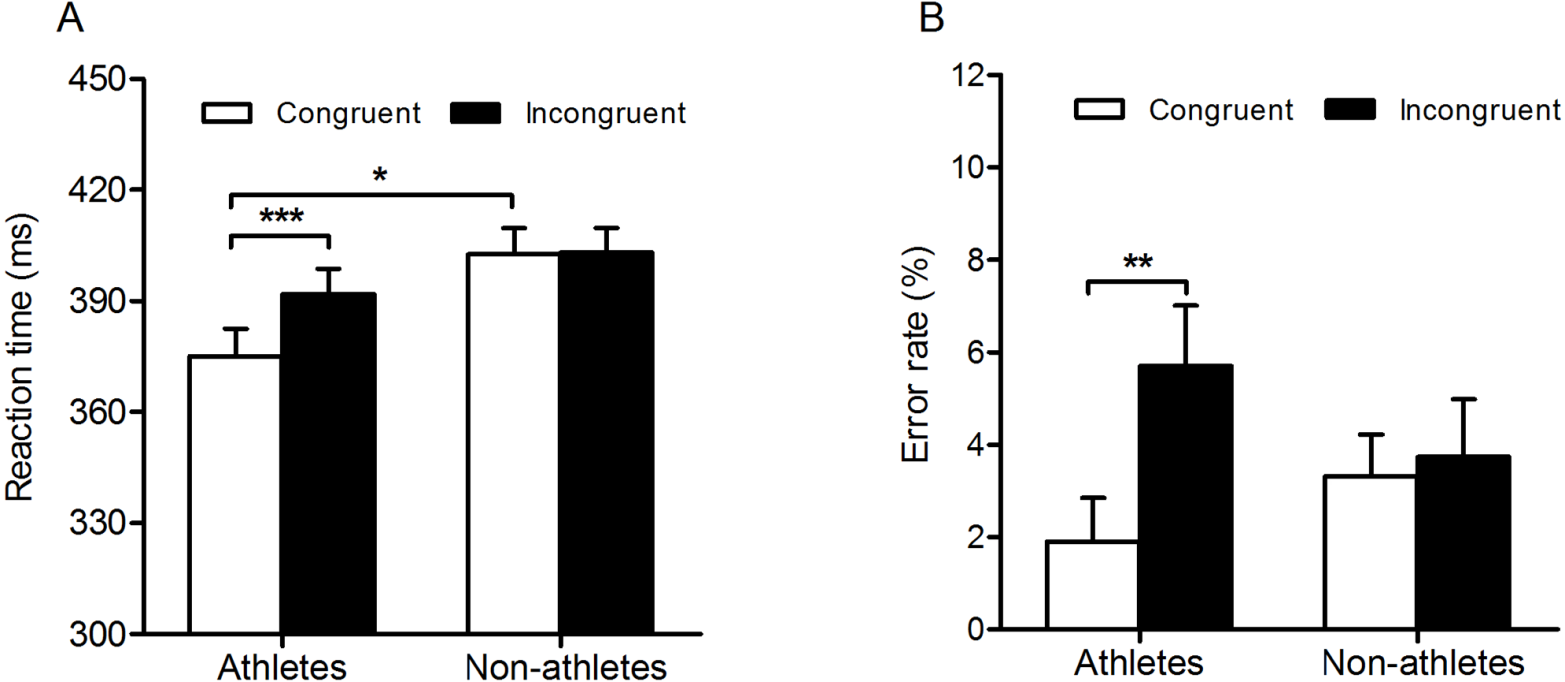
Response times and error rates in the two groups for the unconscious response priming task. Overview of the mean reaction times (A) and error rates (B) for athletes and nonathletes in experiment 1. The vertical bars represent mean reaction times and error rates as a function of response congruency and expertise. White bars represent reaction times and error rates of congruent responses, and black bars represent reaction times and error rates of incongruent responses. The asterisks indicate a statistically significant unconscious response priming effect. Error bars represent standard error of mean (SEM). *p < 0.05; ** *p* < 0.01; *** *p* < 0.001.

The results of two-way repeated measures ANOVAs on RT indicated significant main effects of expertise (*F*_(1,40)_ = 4.21; *p* = 0.047; ${\eta }_{p}^{2}=0.10$) and response congruency (*F*_(1,40)_ = 10.739; *p* < 0.001; ${\eta }_{p}^{2}=0.21$). In addition, there was a significant interaction between expertise and response congruency (*F*_(1,40)_ = 9.61; *p* < 0.001; ${\eta }_{p}^{2}=0.19$). Table tennis athletes responded faster on congruent trials than on incongruent trials (mean ± standard error: congruent, 375.13 ± 7.38 ms; incongruent, 391.94 ±  6.81 ms; *p* < 0.001), whereas no significant difference was found between these two conditions in non-athletes (congruent, 402.69 ± 7.04 ms; incongruent, 403.16 ± 6.49 ms; *p* = 0.90) (Fig. 2A).

The results of a similar ANOVA for error rates indicated a significant main effect of response congruency (*F*_(1,40)_ = 7.23; *p* = 0.01, ${\eta }_{p}^{2}=0.15$). The main effect of expertise did not reach significance. However, the interaction between response congruency and expertise was significant (*F*_(1,40)_ = 4.69; *p* = 0.04; ${\eta }_{p}^{2}=0.11$). Table tennis athletes committed fewer errors on congruent trials than on incongruent trials (congruent, 1.90% ± 0.95%; incongruent, 5.70% ± 1.31%; *p* < 0.001). There was no significant difference in error rates between congruent and incongruent trials for non-athletes (congruent, 3.32% ± 0.90%; incongruent, 3.73% ± 1.25%; *p* = 0.71) (Fig. 2B).

#### Correlation between years of training and unconscious response priming effect in table tennis athletes

**Figure 3:**
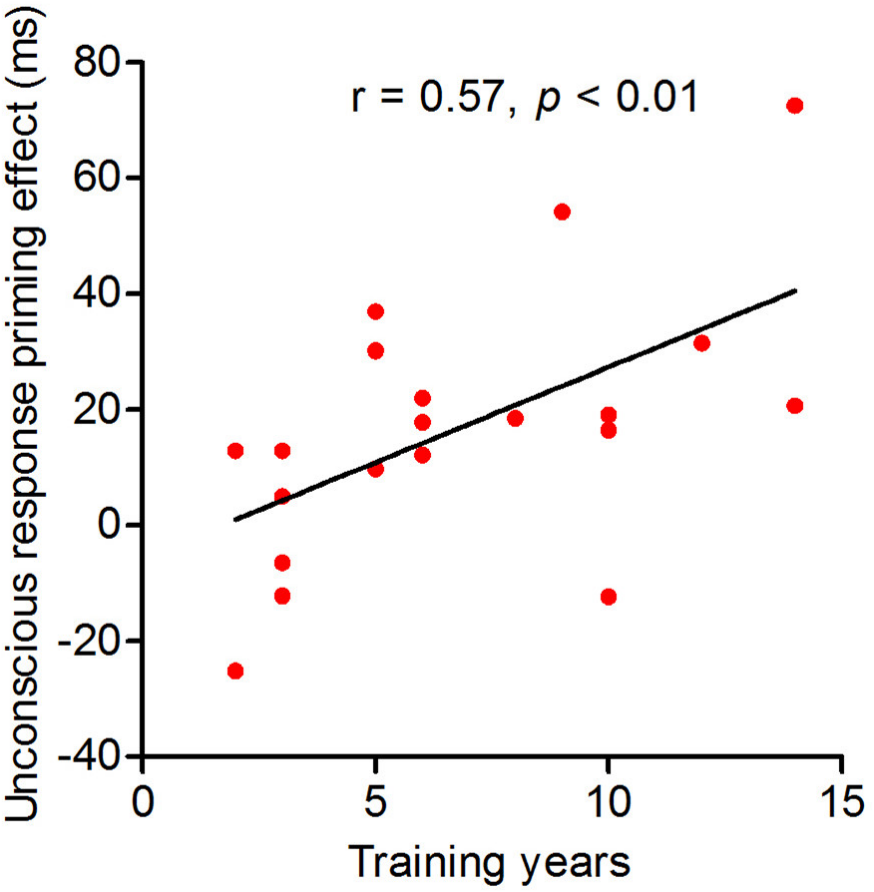
The correlation between the strength of the unconscious response priming effect and number of years of training for table tennis athletes. Correlation between the strength of the unconscious response priming effect and number of years of training in table tennis athletes. The abscissa shows the number of training years, and the ordinate indicates the unconscious response priming effect.

The results of a Pearson’s correlation analysis revealed a significant correlation between the strength of the unconscious response priming effect and the number of years of training (*r*_(20)_ = 0.57, *p* = 0.01). This result indicates that the longer the table tennis athletes had trained, the higher their ability was to unconsciously process visual-spatial information (Fig. 3).

### Discussion for experiment 1

As expected, table tennis athletes showed unconscious response priming, whereas non-athletes did not. This result suggested that table tennis athletes were superior to non-athletes in unconscious processing in executive control. We also found that the number of years the athletes trained was positively correlated with the strength of the unconscious response priming effect. Thus, experiment 1 indicates that motor expertise might modulate the unconscious processing of visual-spatial information in executive control, which is consistent with the findings of Gueldenpenning et al. (2015), Heinemann et al. (2010) that practice or expertise might be a determining factor for unconscious information processing.

## Experiment 2

About half an hour later, we conducted experiment 2 to investigate whether unconscious processing of visual-spatial information in table tennis athletes remains superior to that of non-athletes or whether it is altered when the priming stimuli change from unconscious to conscious. On the basis of previous studies that have suggested that athletes outperform non-athletes in executive control at a conscious level (Chan et al., 2011; Verburgh et al., 2014), we expected stronger response priming in table tennis athletes than in non-athletes. In previous studies, the conscious and unconscious trials were combined and presented within one experiment (Jiang et al., 2016; Ulrich et al., 2013). To avoid the effect of the prime being presented at a conscious level on the unconscious response priming task, the present study required each participant to complete the unconscious response priming task before they started the conscious response priming task (Goller, Khalid & Ansorge, 2017).

### Methods

#### Participants

Participants who took part in experiment 1 also participated in experiment 2.

#### Stimuli

The stimuli used in experiment 2 were identical to those used in experiment 1.

#### Conscious response priming task

The procedure and duration of the conscious response priming task were identical to those in experiment 1 except that (1) the prime was presented for a longer duration, 167 ms (Jiang et al., 2016), and that (2) instead of using a masking stimulus of many randomly oriented lines, blank screens were presented (Kiefer, 2002) (Fig. 1).

### Statistical analysis

#### Reaction times and response errors

The same method as that used in experiment 1 was conducted to reject extreme values (4.9%), and incorrect or missing trials (4.7%) were similarly excluded from further analyses. The mean RT of correct responses and the mean ER were calculated for each participant and each experimental condition. Then, two-way repeated measures ANOVA separately examining mean RT and mean ER were performed using a within-subjects factor of response congruency (congruent vs. incongruent) and a between-subjects factor of expertise (athletes vs. non-athletes).

### Results

#### Reaction times and response errors

**Figure 4:**
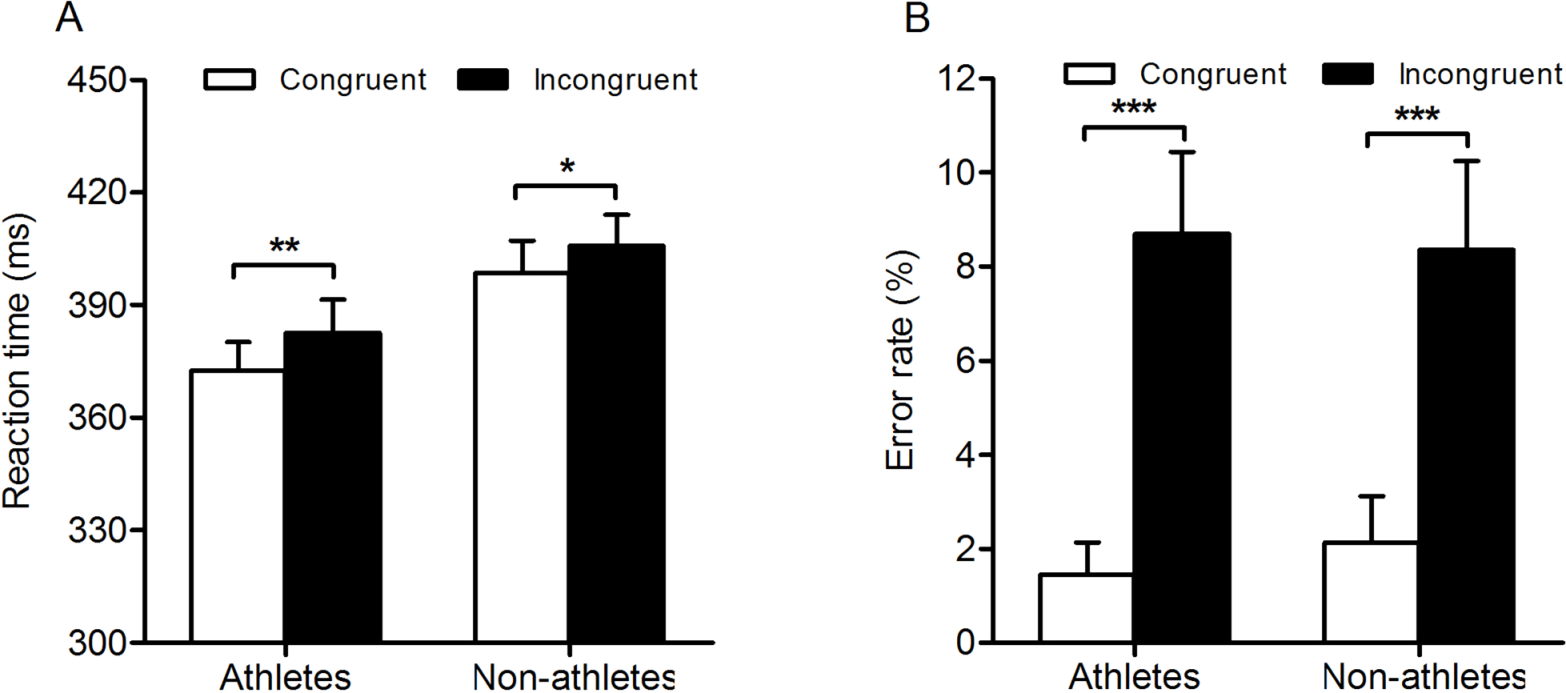
Response times and error rates in the two groups for the conscious response priming task. Overview of the mean reaction time (A) and error rate (B) for athletes and nonathletes in experiment 2. Bars represent the mean reaction times and error rates as a function of response congruency and expertise. White bars indicate reaction times and error rates on congruent trials, and black bars indicate reaction time and error rate on incongruent trials. Error bars represent SEM.

The results of two-way repeated measures ANOVA examining RT showed a significant main effect of expertise (*F*_(1,40)_ = 4.66; *p* = 0.04; ${\eta }_{p}^{2}=0.10$), indicating that the response time in table tennis athletes was much faster than that in non-athletes (athletes, 377.62 ± 8.23 ms; non-athletes, 402.17 ± 7.85 ms). The main effect of response congruency also reached significance (*F*_(1,40)_ = 6.96; *p* = 0.01; ${\eta }_{p}^{2}=0.15$), indicating a faster response on congruent trials than on incongruent trials (congruent, 385.57  ± 5.8 ms; incongruent, 394.22 ± 6.03 ms). However, the interaction between expertise and response congruency did not reach significance (*p* = 0.66) (Fig. 4A).

For the error rates, the main effect of response congruency was significant (*F*_(1,40)_ = 54.31; *p* = 0.00; ${\eta }_{p}^{2}=0.58$), indicating that participants committed fewer errors on congruent trials than on incongruent trials (congruent, 1.79% ± 0.61%; incongruent, 8.53% ±  1.29%). The main effect of expertise (*p* = 0.92) and the interaction between response congruency and expertise did not reach significance (*p* = 0.58) (Fig. 4B).

### Discussion for experiment 2

Consistent with our hypothesis, the results of experiment 2 indicated that table tennis athletes showed a conscious response priming effect. However, non-athletes also showed a conscious response priming effect. These results indicated that the conscious processing of visual-spatial information was independent of motor expertise in present study. This result may be attributable to non-athletes encoding sports information relying on their general experience (Guldenpenning et al., 2012). In addition, the conscious response priming effect in table tennis athletes was not stronger than that in non-athletes, which is in contrast to our hypothesis. The superiority in executive control for table tennis athletes disappeared when the masked (unconscious) prime was changed to an unmasked (conscious) prime. This result, however, may be because the conscious response priming task used in the present study was not too complex to distinguish table tennis athletes from non-athletes.

## General Discussion

The present study investigated whether unconscious executive control in athletes was superior to that in non-athletes and determined whether the superiority of executive control in athletes was affected by the consciousness level. To this end, table tennis athletes and non-athletes took part in unconscious and conscious response priming tasks using circles with notches at one of four positions as primes and targets. Our primary results showed that table tennis athletes responded faster and committed fewer errors on congruent trials than on incongruent trials, whereas no significant difference between these two conditions was found in non-athletes at unconscious level. Furthermore, there was a positive correlation between the number of years of training and the strength of the unconscious response priming effect in table tennis athletes. However, both table tennis athletes and non-athletes responded faster and committed fewer errors on congruent trials than on incongruent trials when the primes were changed from being masked (at the unconscious level) to being at the conscious level. Moreover, no significant difference was found between table tennis athletes and non-athletes for this conscious response priming effect.

### Motor expertise modulates unconscious executive control

In experiment 1, table tennis athletes showed an unconscious response priming effect, whereas non-athletes did not, which is consistent with the findings of previous studies that expertise and practice may be a prerequisite for the unconscious processing of domain-specific stimuli (Heinemann et al., 2010; Kiesel et al., 2009; Reuss et al., 2015). Perceptual sensitivity (Gueldenpenning et al., 2011) and perceptual-motor common representations (Hommel et al., 2001) might explain the superiority of unconscious processing in executive control that was found among table tennis athletes versus non-athletes.

On the one hand, the stimuli used in the present study, circles with a notch at one of four positions, were designed based on the item characteristics of table tennis. Our findings suggest that table tennis athletes exhibit higher perceptual sensitivity to the circles with angles in comparison with non-athletes owing to extensive table tennis training (Guo, Li & Yu, 2017). The stimuli used in the present study differed from those used in previous research, such as digital stimuli (Greenwald, Draine & Abrams, 1996), faces showing emotions (Duan et al., 2010), easy geometries (Ulrich & Kiefer, 2016), and words (Ortells et al., 2016) that are well known to almost everyone. This result is consistent with the findings that compared to non-athletes, athletes with specific motor experience exhibit higher perceptual sensitivity for stimuli in their domain (Schütz-Bosbach & Prinz, 2007). Notably, Gueldenpenning et al. (2011) suggested that the high perceptual sensitivity might be the prerequisite to unconscious processing. Thus, judging the notch orientation was considered an expert task for table tennis athletes and a non-expert task for non-athletes, and revealing a motor expertise modulation of unconscious processing (Gueldenpenning et al., 2015).

On the other hand, the two-stage process that was put forward by Kibele (2006) based on the theory of event coding also well explains the superiority of unconscious processing in executive control in table tennis athletes. The first stage is learning, in which the unconscious associations between the perceived information and corresponding reactions are established, and a lot of practice is essential at this stage. The second stage is performance. When individual unconsciously perceived the information, the perceptual-motor representations established previously are extracted; then a response is pre-activated. Considering that the flying speed of the ball is fast and the rotation of the ball is changeable in table tennis, and opponents usually perform a fake when the racket batted the ball. Consequently, table tennis athletes need to quickly make judgments based on continuously updated information. For table tennis athletes, the unconsciously perceived visual-spatial information could activate the previously acquired perceptual-motor representations about table tennis and therefore prime a motor response. If the pre-activated response is in accordance with a required response to a target, the response would be facilitated and the response error would be reduced. If the pre-activated response is inconsistent with a required response to a target, table tennis athletes need to inhabit the inappropriate response tendency, and then change the incorrect task set induced by the cued information presented previously and timely retrieve the perceptual-motor representations again. However, the response would be interfered and committed more errors under this condition. Due to lack of practical motor expertise in table tennis, non-athletes could not improve their performance in the present study through perceptual-motor representations.

However, another study conducted by Gueldenpenning et al. (2011) with unconscious priming task that differed from our findings. In their task, the prime and target were photographs of high jump movement, and high jump movement was divided into approach phase and flight phase. Participants were told to determine which part of a high jump that target depicted. The results found that both athletes and non-athletes did not present the response congruency effect. The lack of response congruency effect might be attributed to the experiment design. In addition to the motor expertise and response congruency, they also selected the temporal order of movement as between subject variable. When the prime depicted a movement segment that is earlier in time than the target, it was called natural temporal order, or else, it was called reversed temporal order. It is assumed that the processing of temporal order might weaken the processing of movement phases. Fine-tuned movement control in every step was the main characteristic of high jump. Compared to the movement phases, unconscious processing of temporal order information might better reflect the motor expertise that high jump athletes possess. Thus, the modulatory effect of motor expertise on unconscious processing in executive control might only existed in the processing of key information.

In addition, the number of years of training for the athletes was positively correlated with the strength of the unconscious response priming effect. The more training table tennis athletes received, the higher perceptual sensitivity they possessed. Meanwhile, the automatic associations between perceptual and motor responses would be enhanced with the accumulation of motor experience. This finding is consistent with that from the study of Weissensteiner, Abernethy & Farrow (2011), who found that the performance of highly skilled cricket batsmen is better than that of lesser skilled cricket batsmen because of stronger perception-action coupling.

On the basis of the previous findings that the dorsal stream enables sensory information and response parameters to be linked directly without the mediation of consciousness (Goodale & Milner, 1992; James et al., 2003; Gueldenpenning et al., 2011) also assumed that unconscious movement processing in athletes might rely on this dorsal pathway. However, the unconscious processing found in sports scenarios relies on two control systems: off-line control and on-line control. The off-line control is more sensitive to conscious intentions, which corresponds to the ventral pathway. The on-line control can activate a response based on the most up-to-date unconscious information, which corresponds to the dorsal pathway (Hommel, 2003). Furthermore, recent research found that both the dorsal and ventral pathways can affect unconscious information processing (Ulrich & Kiefer, 2016). Thus, unconscious information processing in executive control among table tennis athletes might be interpreted from the perspective of an interaction between the dorsal and ventral streams.

### Conscious executive control is independent of motor skill experience

In experiment 2, both table tennis athletes and non-athletes responded faster and committed fewer errors on congruent trials than on incongruent trials. Hence, both showed conscious response priming effects. Each participant could consciously perceive the prime when the masked prime changed to an unmasked prime. For table tennis athletes, the perceptual-motor representations were activated by the conscious processing of visual-motor information similar to that in the unconscious condition. This may result in reaction promotion on congruent trials but reaction confliction on incongruent trials. For non-athletes, the stimuli of circles with a notch were viewed merely as incomplete geometries having a direction, like arrows, and their direction indicated the correct response side. The general experience of non-athletes might have been sufficient for encoding the prime and then producing a pre-activated response. This interpretation is in line with a previous study, which suggested that general motor experience helps non-athletes form a rough movement prediction (Guldenpenning et al., 2012).

Contrary to our hypothesis, there was no significant difference between the two groups in the conscious response priming effect. Table tennis athletes did not show superiority in executive control when the primes were perceived consciously. This result indicates that motor expertise fail to modulate conscious executive control. This is similar with some studies that performance benefits on cognitive in favor of athletes were not found (Chang et al., 2017; Lum, Enns & Pratt, 2002). A meta-analytic review have indicated that age, gender, level of expertise and the type of cognitive task were the potential moderator variables of exercise effects on cognition (Voss et al., 2010). In present study, we speculate that the conscious response priming task may have been not too complex to distinguish table tennis athletes from non-athletes. In addition, given a meta-analytic review found the brain areas that are associated with executive control were only activated under complex cognitive tasks, and suggested that executive control was task-dependent (Simmonds, Pekar & Mostofsky, 2008). Therefore, our findings suggested that the difficulty level of the cognitive task might be another moderator that affected the positive relationship between expertise and cognition.

When the visibility of the prime changed from conscious to unconscious, the priming effect disappeared in non-athletes. This result suggests that although non-athletes could process the visual-spatial information offered by the primes on a conscious level, they could not process it on an unconscious level. Whereas, no matter how the visibility of the prime changed, the priming effect always existed in table tennis athletes. The present study found that the unconscious processing in executive control may better reflect the superiority in executive control, and indicate that motor skill experience modulates unconscious, rather than conscious, executive control in table tennis. The unconscious processing in executive control may facilitate adaptation to the rapid and complex changing contexts, which may be the key factor in determining the outcome of table tennis competitions. Thus, the unconscious processing in executive control is critical for table tennis athletes. In addition, compared to conscious executive control, the unconscious processing in executive control might also be used to assess the level of executive control for table tennis athletes.

## Limitations

The present study had a few limitations. Firstly, we explained the pre-activated response triggered by conscious and unconscious priming at the behavioral level. Event-related potentials are a more direct and objective technique, and the lateralized readiness potential component can be used to evaluate response preparation in future research (Dehaene et al., 1998; Leuthold & Kopp, 2010). Secondly, the present results indicated that there might be a positive correlation between the number of years of training and the strength of the unconscious response priming effect. A longitudinal intervention experimental design may help demonstrate a causal relationship between these two factors. Thirdly, the number of participants in present study was relatively small. Thus, individual differences may have contributed to our results. A larger sample of participants should be recruited in future studies to remove the effect that individual differences may have had in the present study. Finally, all the athlete participants were from table tennis, another group of athletes who did not share the specific characteristics with table tennis (e.g., rugby, badminton) should be added in future study to better prove the modulatory effect of motor expertise on unconscious executive control among table tennis athletes.

## Conclusions

Table tennis athletes, but not non-athletes, demonstrated an unconscious response priming effect that was positively correlated with motor expertise. However, both table tennis athletes and non-athletes showed similar conscious response priming effects. These findings provide supporting evidence that motor expertise modulates unconscious rather than conscious executive control in a priming task and suggest that unconscious information processing may underlie the executive control superiority observed in table tennis athletes versus non-athletes.

##  Supplemental Information

10.7717/peerj.6387/supp-1 Supplemental Information 1 Reaction times and response errors for conscious priming task

10.7717/peerj.6387/supp-2 Supplemental Information 2 Reaction times and response errors for unconscious priming task

## Additional Information and Declarations

### Data Availability

The following information was supplied regarding data availability: Raw data are available in the Supplemental Files.
